# Gastric cancer‐associated long non‐coding RNA profiling and noninvasive biomarker screening based on a high‐risk population cohort

**DOI:** 10.1002/cam4.5905

**Published:** 2023-04-21

**Authors:** Xiaoying Guo, Yang Zhang, Zhiyi Zhang, Linzhi Lu, Yuqin Liu, Zhexuan Li, Tong Zhou, Jingying Zhang, Wenqing Li, Weicheng You, Guoquan Tao, Wanqing Chen, Hongmei Zeng, Kaifeng Pan

**Affiliations:** ^1^ Key Laboratory of Carcinogenesis and Translational Research (Ministry of Education/Beijing), Department of Cancer Epidemiology, Peking University School of Oncology Peking University Cancer Hospital & Institute Beijing China; ^2^ Department of Gastroenterology Gansu Wuwei Tumor Hospital Wuwei China; ^3^ Cancer Epidemiology Research Center Gansu Provincial Cancer Hospital Lanzhou China; ^4^ Department of General Surgery, The Affiliated Huai'an No.1 People's Hospital Nanjing Medical University Huai'an 223300 China; ^5^ National Cancer Center/National Clinical Research Center for Cancer/Cancer Hospital Chinese Academy of Medical Sciences and Peking Union Medical College Beijing China

**Keywords:** biomarkers, gastric cancer, LncRNAs, microarray, plasma

## Abstract

**Background:**

Effective noninvasive biomarkers of gastric cancer (GC) are critical for early detection and improvement of prognosis. We performed genome‐wide long non‐coding RNA (lncRNA) microarray analysis to identify and validate novel GC biomarkers depending on a high‐risk population cohort.

**Methods:**

LncRNA profiles were described using the Human LncRNA Microarray between GC and control plasma samples. The differential candidate lncRNAs were validated in two stages by quantitative reverse transcription polymerase chain reaction (qRT‐PCR). We further evaluated the joint effect between the GC‐associated lncRNA and *Helicobacter pylori* (*H. pylori*) infection on the risk of cardia and non‐cardia GC, respectively.

**Results:**

Different lncRNA expression profiles were identified between GC and control plasma with a total of 1206 differential lncRNAs including 470 upregulated and 736 downregulated in GC compared with the control group. The eight significantly upregulated lncRNAs (*RP11‐521D12.1*, *AC011995.3*, *RP11‐5P4.3*, *RP11‐244 K5.6*, *RP11‐422 J15.1*, *CTD‐2306 M5.1*, *CTC‐428G20.2*, and *AC009133.20*) in GC cases both in the present study and a similar microarray screening study by our collaborative team were selected for a two‐stage validation. After the large sample size validation, the subjects with higher expression of *RP11‐244 K5.6* showed a significantly increased risk of GC with an adjusted odds ratio (OR) as 2.68 and 95% confidence interval (CI) as 1.15–6.24. Joint effects between *RP11‐244 K5.6* expression and *H. pylori* infection on the risk of GC were evaluated with no statistical significance.

**Conclusions:**

Our study found different lncRNA expression profiles between GC and control plasma and preliminarily identified *RP11‐244 K5.6* as a potential noninvasive biomarker for GC screening.

## Graphical abstract

Lnc RNA expression profiles between gastric cancer and control plasma were compared using the Human LncRNA Microarray with *RP11‐244 K5.6* as a potential noninvasive biomarker.

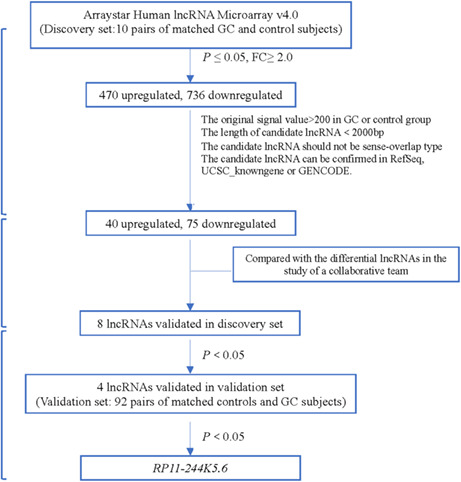

## 1 INTRODUCTION

Gastric cancer (GC) is the fifth most common cancer and the third leading cause of cancer death worldwide.^1^ Over 40% of new cases and deaths of GC occur in China, which brings huge economic and social burdens.^2^ The prognosis of GC can be improved dramatically by early detection.^3^ However, effective and sensitive early detection biomarkers are currently unavailable due to the unclear complex gene regulation network in the occurrence process of GC.^4^, ^5^

The Human Genome Project revealed that more than 98% of the genome is transcribed into non‐coding RNAs (ncRNAs).^6^ According to the length of ncRNAs, long non‐coding RNAs (lncRNAs) are a transcript RNA molecule with a length of more than 200 nts.^7^ Accumulating studies have confirmed that lncRNAs are widely involved in gene expression, epigenetic regulation, transcription, and other vital physiology processes.^8^, ^9^, ^10^ Moreover, recent findings suggest that the aberrant expression of lncRNAs can be stably detected in the plasma samples of many kinds of cancer subjects, such as lung cancer, breast cancer, and colorectal cancer.^11^, ^12^, ^13^, ^14^ LncRNAs may serve as potential noninvasive biomarkers for GC, although systematic investigations are still needed.

In addition to the lifestyle factors and nutrient supplementation,^15^ *Helicobacter pylori* (*H. pylori*) infection is one of the most important risk factors for GC. The pathogenesis of *H. pylori* may involve a complicated combination of host genetics, bacterial virulence, and environmental agents.^16^ A study analyzed GC cases from TCGA database and identified differential lncRNAs in *H. pylori*‐positive GC cases compared with negative ones, which suggested possible roles of lncRNAs in *H. pylori*‐associated carcinogenesis.^17^ However, the interactions between *H. pylori* infection and lncRNAs on the risk of GC remain unclear.

Most of the previous studies on GC‐associated lncRNAs and their biological roles were conducted in cancer cell lines or hospital‐sourced patients. A population‐based cohort study will be more promising for lncRNA biomarker screening. In the present study, we compared the expression profiles of lncRNAs between paired GC and control plasma using a high throughput updated Human LncRNA Microarray. Novel differential lncRNA biomarkers were selected and validated depending on an endoscopic screening cohort from a high‐risk population in Wuwei County, Gansu Province, China. Furthermore, the interactions between lncRNA biomarkers and possible influence factors, such as *H. pylori* infection, smoking, and drinking, in the gastric carcinogenesis process were also investigated.

## 2 MATERIALS AND METHODS

### 2.1 Study population

**FIGURE 1:**
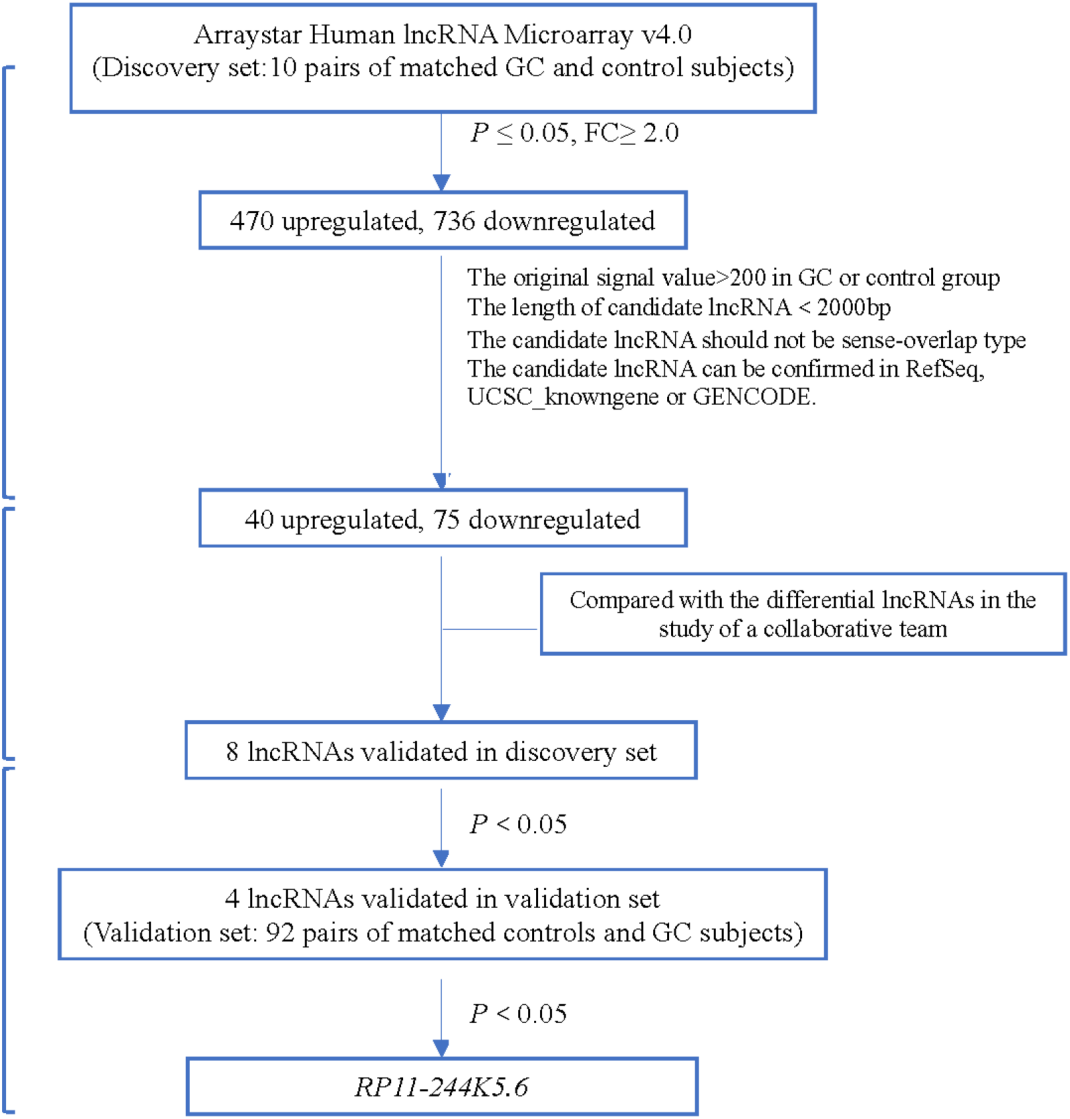
Flow process of study design.

The present study was conducted within the framework of the National Upper Gastrointestinal Cancer Early Detection Project in Wuwei County, Gansu Province of China, one of the high‐risk areas of upper gastrointestinal cancer. GC cases and non‐GC controls matched by age and gender were selected from the endoscopic screening participants from January 2015 to February 2016. All GC cases were pathologically confirmed by WHO standard.^18^ Plasma samples were collected in preliminary screening before any clinical therapy and characteristic information was obtained by a standard structured questionnaire including sex, age, smoking and drinking habits. In the lncRNA microarray analysis and small sample size validation, 10 pairs of GC and matched control subjects were enrolled as a discovery set to screen and preliminarily select candidate differential lncRNAs. For large sample size validation using quantitative reverse transcription polymerase chain reaction (qRT‐PCR), the preliminarily selected candidate lncRNAs were further compared between 92 GC cases and 184 matched controls at a ratio of 1:2 in a validation set (Figure 1). All of the 296 subjects have provided written informed consent. This study was approved by the Institutional Review Boards of the Chinese Academy of Medical Sciences Cancer Hospital (Approval No. 18–015/1644).

### 2.2 Blood sample processing

From each subject, a 10 mL peripheral blood sample was collected into EDTA‐treated tubes (BD Vacutainer) and centrifuged within 30 min after collection at 3000*g* for 10 min to separate plasma. The plasma was then stored at −80°C until detection.

### 2.3 RNA extraction and reverse transcription

Total RNA was extracted from 400 μL of plasma using miRNeasy Serum/plasma Kit (Qiagen, Germany) following the manufacturer's instructions. Total RNA was extracted from cultured cells using TRIzol reagent (Invitrogen, USA) according to the manufacturer's protocol. SuperScript™ III First‐Strand Synthesis SuperMix (Invitrogen, USA) was used to generate cDNA in qRT‐PCR.

### 2.4 LncRNA microarray detection

The Arraystar Human lncRNA Microarray v4.0 (Aksomics, China) was used to compare the lncRNA expression profiles between ten GC cases and matched controls. As an updated version from the previous Microarray v3.0 (covering 30,586 human lncRNAs), the Microarray v4.0 can detect 40,916 human lncRNAs. The microarray detection and data collection were conducted by KangChen Biotech (Shanghai, China). Briefly, the array images were obtained and analyzed by Agilent Feature Extraction software after total RNA extraction, reverse transcription, microarray hybridization, slide washing, and microarray scanning. Quantile normalization and subsequent data processing were carried out using the R software package.

### 2.5 The evaluation of internal controls for the qualification of plasma lncRNAs

According to the previous studies, three frequently used reference genes (*GAPDH*, *ß‐actin*, and *U6*) were evaluated in the discovery set (including 10 pairs of age‐ and sex‐matched GC and control subjects). NormFinder, which is one of the algorithms for reference gene selection, was used to evaluate both intra‐ and inter‐group variations between GC and control groups. For the three reference genes, the values of the stability were calculated and compared.

### 2.6 Quantitative real‐time PCR

Quantitative real‐time PCR was performed using the Arraystar SYBR® Green qPCR Master Mix (ROX+) (Arraystar, USA) on an Applied Biosystems 7500 FAST Real‐time PCR system (Applied Biosystems, USA). Relative quantification of target gene expression was calculated with 2^−ΔΔCT^ method by dividing the target lncRNA/reference gene ratio of a tested plasma sample by the target lncRNA/reference gene ratio of a reference sample. For the small sample size validation in the discovery set, we used a pooled sample from the ten GC subjects as the reference sample. In the large sample size validation, the RNA extracted from the human gastric cell line MGC‐803 was used as the reference sample on every PCR detection plate with all samples assayed in duplicate. The MGC‐803 cell line was gifted from the Laboratory of Biochemistry and Molecular Biology, Peking University Cancer Hospital & Institute. The samples with the Ct value for a candidate lncRNA <36 are considered positive expression. All the primers for candidate lncRNAs or reference genes were synthesized by Augct Biotech (Beijing, China), and the sequences are shown in Table S1.

### 2.7 Measurement of *H. pylori* infection status

As reported previously, we used *H. pylori* antibody assays to determine the *H. pylori* infection status.^19^ Briefly, Enzyme‐Linked Immunosorbent Assay (ELISA) was used to measure plasma levels of anti‐*H. pylori* IgG by duplicate procedures. The mean optical density for IgG ≥1.0 was defined to be positive for *H. pylori* infection.

### 2.8 Statistical analysis

A paired *t*‐test was conducted to identify the differential lncRNAs between GC and control plasma samples according to the criteria of fold change (FC) ≥ 2.0 and *p*‐value ≤0.05 in microarray analysis and small sample size validation. Furthermore, hierarchical clustering analysis and volcano plot were performed to illustrate different gene expression patterns. NormFinder software (https://www.moma.dk/normfinder‐software/, NormFinder for R, version 5 2015‐01‐05) was carried out by R software (version 3.5.2) to identify the stability of candidate reference genes. The gene with the highest stability value was selected as the optimal reference gene for the quantitative analysis of candidate lncRNAs. For large sample size validation, the Pearson's *χ* ^*2*^ test was used to compare the differences in sex, *H. pylori* infection status, and smoking and drinking habits between GC cases and controls. The lncRNA expression levels were categorized into low and high expression status with the expression medians of candidate lncRNAs in the control group as cutoff values. Conditional logistic regression was used to evaluate the associations of candidate lncRNA expression status with the risk of GC adjusted for smoking and drinking habits. For the joint effects between lncRNA expression and possible influence factors, including *H. pylori* infection, and smoking and drinking habits, unconditional logistic regression was conducted adjusted for age, gender, and other confounding factors. Statistical analyses were performed using SPSS version 24.0 (SPSS) and graphically presented by GraphPad Prism 5.0. Two‐tailed *p*‐values of <0.05 were considered statistically significant.

## 3 RESULTS

### 3.1 General characteristics of the study subjects

The discovery set for microarray screening and small sample size validation contained 10 pairs of age‐ and gender‐matched GC and control subjects. The mean age was 60.3 ± 10.9 years in the GC group and 60.3 ± 10.0 years in the control group. Both of the two groups contained 7 males and 3 females. There were 5 *H. pylori*‐positive cases in the GC group and 4 positive subjects in the control group, respectively.

The general characteristics of the validation set were shown in Table 1. For each GC case, two age‐ and gender‐matched controls were selected. There were no significant differences in mean age, gender, and smoking and drinking habits between the GC and control groups. However, the infection rate of *H. pylori* was statistically higher in the GC group compared with the control group (63.0% vs. 47.3%, *p* = 0.016). Among GC subjects, 19 cases (20.7%) were diagnosed as high‐grade intraepithelial neoplasia (HGIN) and 73 cases (79.3%) were advanced GC according to WHO standard. A total of 34 GC (37.0%) were located in cardia and 58 GC (63.0%) were located in non‐cardia.

**TABLE 1:** General characteristics of the subjects in validation set

Characteristics	Cases (*n* = 92) (%)	Controls (*n* = 184) (%)	*p*
Age (years)			0.922^a^
Mean ± SD	59.1 ± 7.2	58.7 ± 7.1	
Sex, No (%)			
Female	21 (22.8)	42 (22.8)	0.932^b^
Male	71 (77.2)	142 (77.2)	
*H. pylori* infection^a^			
Positive	58 (63.0)	87 (47.3)	0.016^b^
Negative	34 (37.0)	97 (52.7)	
Smoking			
Ever or current	55 (59.8)	105 (57.1)	0.666^b^
Never	37 (40.2)	79 (42.9)	
Drinking			0.103^b^
Ever or current	26 (28.3)	36 (19.6)	
Never	66 (71.7)	148 (80.4)	
Stage			
HGIN	19 (20.7)		
Cancer	73 (79.3)		
Location			
Cardia	34 (37.0)		
Non‐cardia	58 (63.0)		

^a^ Student's *t*‐test.

^b^ Pearson *χ* ^*2*^ test.

### 3.2 The results of lncRNA microarray analysis and candidate lncRNA selection

**FIGURE 2:**
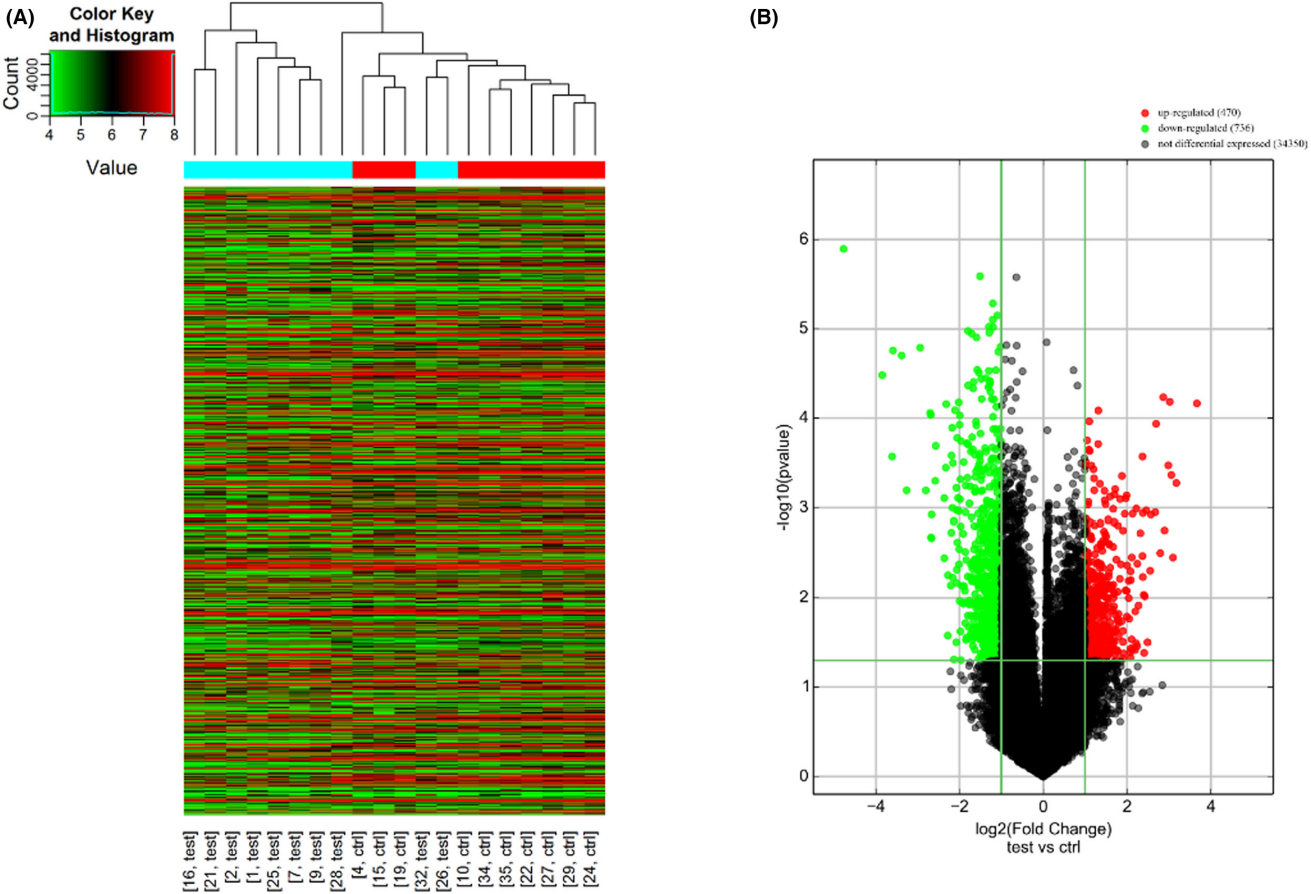
LncRNA expression profiles comparison between GC and control groups by microarray analysis. (A) Hierarchical clustering visualized the differential expression of lncRNAs among subjects. Each row represents the expression level of a differential lncRNA. Each column represents a plasma sample from each subject (test—gastric cancer plasma sample; ctrl—control plasma sample). (B) The volcano plot showed the differential expression of lncRNAs. Red dots represent upregulated lncRNAs and green dots represent downregulated lncRNAs in gastric cancer plasma (Fold change≥2 and *p* ≤ 0.05).

The lncRNA expression profiles were compared between ten GC and ten control subjects in the discovery set. Hierarchical clustering visualized the differential expression levels of lncRNAs between GC and control groups (Figure 2A). The volcano plot showed a total of 1206 differential lncRNAs (FC ≥2.0 and *p*‐value ≤0.05) including 470 upregulated and 736 downregulated lncRNAs in GC compared with control plasma samples (Figure 2B).

In order to investigate potential candidate lncRNAs for noninvasive biomarker screening, we selected differential lncRNAs in microarray analysis for small sample size validation according to the following criteria. (1) The original signal value should be more than 200 in the GC group or control group. (2) The length of the candidate lncRNA should be less than 2000 bp. (3) The sense‐overlapping lncRNAs, which are difficult to be separated from the overlapping protein‐coding genes in sense orientation, should not be selected. (4) The candidate lncRNAs can be confirmed in the databases of RefSeq, UCSC_knowngene, or GENCODE. A total of 40 upregulated and 75 downregulated lncRNAs were selected according to the four criteria.

In addition, the candidate upregulated and downregulated lncRNAs in the present study were further compared with the differential lncRNAs in a similar microarray study using Arraystar Human lncRNA Microarray v3.0 (Aksomics, China) between five GC cases and matched healthy controls by our collaborative team.^20^ Finally, eight upregulated candidate lncRNAs in GC cases of the two studies were enrolled into the small sample size validation stage, including *RP11‐521D12.1* (ENST00000478468), *AC011995.3* (ENST00000437610), *RP11‐5P4.3* (ENST00000427547), *RP11‐244 K5.6* (ENST00000435483), *RP11‐422 J15.1* (ENST00000507099), *CTD‐2306 M5.1* (ENST00000507887), *CTC‐428G20.2* (ENST00000514544), and *AC009133.20* (ENST00000569039).

### 3.3 The evaluation of internal controls for the quantitative analysis of candidate lncRNAs

Because the previous quantitative analyses of lncRNAs used different internal control genes, the present study evaluated three frequently used internal control genes including *U6*, *GAPDH*, and *ß‐actin*. The stability comparison of the three candidate reference genes in the discovery set found that the stability values were increased from 0.09 for *ß‐actin* to 0.22 for *GAPDH* and 0.80 for *U6* genes (Figure S1). Consequently, *U6* gene was selected as the optimal reference gene for the subsequent validations of candidate lncRNAs in plasma samples.

### 3.4 Small sample size validation of the candidate lncRNAs from microarray screening

**FIGURE 3:**
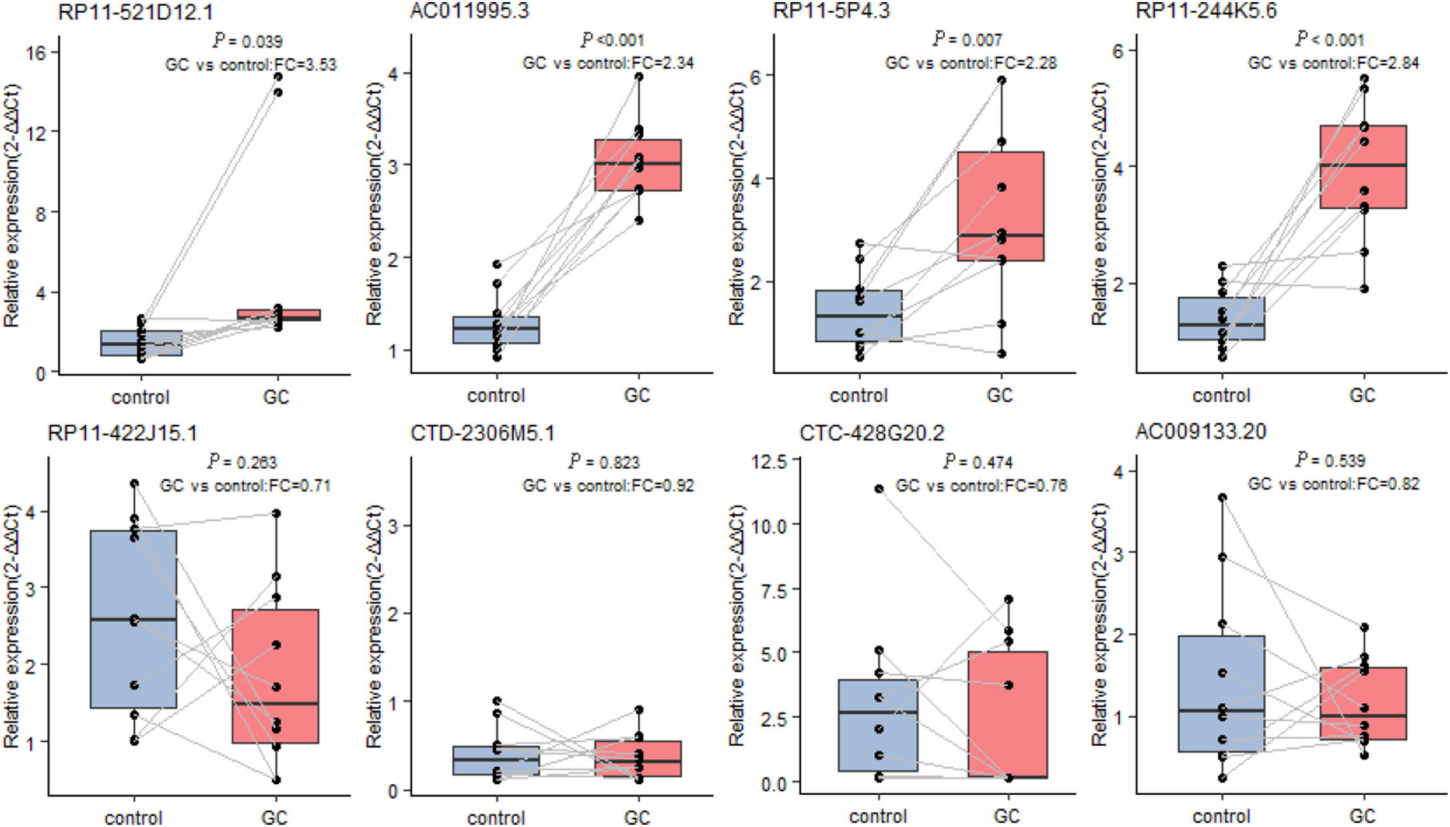
Small sample size validation of the 8 candidate lncRNAs. A paired *t*‐test was conducted to compare the 8 candidate lncRNAs between GC and control plasma samples. The differential lncRNAs were selected according to the criteria of fold change (FC) ≥ 2.0 and *p*‐value ≤0.05.

The selected eight candidate upregulated lncRNAs were validated by qRT‐PCR in the same discovery set as microarray screening with the ten GC cases and 10 controls. The expression levels of four lncRNAs were increased significantly in the plasma samples of GC cases compared with control subjects, including *RP11‐521D12.1*, *AC011995.3*, *RP11‐5P4.3* and *RP11‐244 K5.6* (all *p‐*values <0.05) (Figure 3).

### 3.5 Large sample size validation of the candidate lncRNAs

The four significant lncRNAs in the small sample size validation were further compared in the 92 GC cases and 184 matched controls at a ratio of 1:2. The candidate lncRNA was considered positively expressed in plasma when the Ct value was less than 36 by qRT‐PCR detection. The positive expression rates were 91.0% for *RP11‐521D12.1*, 73.9% for *AC011995.3*, 97.8% for *RP11‐5P4.3*, and 93.5% for *RP11‐244 K5.6* in the GC group. The positive rates of the four lncRNAs in the GC group showed no significant differences compared with those of the control group (90.2%, 71.7%, 92.4%, and 87.5%) with all *p*‐values >0.05.

The expression levels of the four candidate lncRNAs were divided into high and low expression status with the medians of the expression levels in the control group as cutoff values. The subjects with high expression status of *RP11‐244 K5.6* showed an increased risk of GC with an adjusted odds ratio (OR) as 2.68 and 95% confidence interval (95%CI) as 1.15–6.24 (Table 2). However, the expression status of the other three candidate lncRNAs showed no significant differences in GC and control groups (all *p*‐values >0.05).

**TABLE 2:** Large sample size validation of the candidate lncRNAs.

LncRNA expression status	GC Cases *n* (%)	Controls *n* (%)	OR (95%CI)	*p* ^a^
RP11‐521D12.1				
Low	41 (48.8)	83 (49.7)	1.00	
High	43 (51.2)	84 (50.3)	1.12 (0.54–2.30)	0.766
AC011995.3				
Low	38 (55.9)	66 (50.0)	1.00	
High	30 (44.1)	66 (50.0)	0.60 (0.24–1.49)	0.268
RP11‐5P4.3				
Low	41 (45.6)	85 (50.0)	1.00	
High	49 (54.4)	85 (50.0)	1.37 (0.73–2.57)	0.327
RP11‐244 K5.6				
Low	34 (39.5)	81 (50.0)	1.00	
High	52 (60.5)	80 (50.0)	2.68 (1.15–6.24)	0.022

^a^ Conditional logistic regression adjusted for age, sex, smoking, and drinking.

### 3.6 Joint effects between lncRNA expression and possible influence factors on risk of GC

Because *H. pylori* infection frequency was higher in the GC group, it might act as an important risk factor for gastric carcinogenesis in our study population. *H. pylori* infection status was also considered in the combination of *RP11‐244 K5.6* expression on GC risk. Analysis of joint effects found that the subjects with only high expression status of *RP11‐244 K5.6* (OR, 2.74, 95%CI: 1.15–6.54) or the subjects with only *H. pylori*‐positive status (OR, 3.50, 95%CI: 1.46–8.41) both showed a higher risk of GC compared to the subjects with negative *H. pylori* status and low expression of *RP11‐244 K5.6*. Although the subjects with both positive *H. pylori* infection and high expression status of *RP11‐244 K5.6* also showed a higher risk of GC (OR, 3.60, 95%CI: 1.54–8.43), no significant joint effect was found between *H. pylori* infection and *RP11‐244 K5.6* expression on GC risk with *p*_interaction_ = 0.094 (Table 3).

**TABLE 3:** Joint effects between *RP11‐244 K5.6* expression levels and *H. pylori* infection on risk of GC.

LncRNA status	*H. pylori* infection	GC Cases *n* (%)	Controls *n* (%)	OR (95%CI)	*p* ^a^
Low	Negative	10 (11.6)	47 (29.2)	1.00	
Low	Positive	24 (27.9)	34 (21.1)	3.50 (1.46–8.41)	0.005
High	Negative	22 (25.6)	39 (24.2)	2.74 (1.15–6.54)	0.023
High	Positive	30 (34.9)	41 (25.5)	3.60 (1.54–8.43)	0.003

^a^ Unconditional logistic regression adjusted for age, sex, smoking, and drinking.

We further conducted stratified analysis by classifying GC cases into cardia and non‐cardia cancer. The subjects with the high expression of *RP11‐244 K5.6* and *H. pylori*‐positive status showed a significantly higher risk of cardia GC (OR, 4.04, 95%CI: 1.03–15.85) compared to *H. pylori*‐negative subjects with the low expression of *RP11‐244 K5.6*. However, no statistical significance was found for those with only *H. pylori*‐positive status (OR, 3.55, 95%CI: 0.84–14.90) or for those with only high expression status of *RP11‐244 K5.6* (OR, 3.22, 95%CI: 0.78–13.25). On the other hand, the subjects with the high expression of *RP11‐244 K5.6* and *H. pylori*‐positive status showed a higher risk of non‐cardia GC (OR, 3.33, 95%CI: 1.24–8.99) compared to *H. pylori*‐negative subjects with the low expression level of *RP11‐244 K5.6*. Although we found a higher risk of non‐cardia cancer for the cases with only *H. pylori*‐positive status (OR, 3.52, 95%CI: 1.28–9.65), no statistical significance was found for those with only the high expression of *RP11‐244 K5.6* (Table 4). However, no significant joint effects were found between *H. pylori* infection and *RP11‐244 K5.6* expression on cardia GC risk (*p*_interaction_ = 0.319) or non‐cardia GC risk (*p*_interaction_ = 0.141).

**TABLE 4:** Joint effects between *RP11‐244 K5.6* expression and *H. pylori* infection status in cardia and non‐cardia GC.

GC	LncRNA expression status	*H. pylori* infection	GC Cases *n* (%)	Controls *n* (%)	OR (95%CI)	*p* ^a^
Cardia	Low	Negative	3 (9.7)	47 (29.2)	1.00	
	Low	Positive	8 (25.8)	34 (21.1)	3.55 (0.84–14.90)	0.084
	High	Negative	8 (25.8)	39 (24.2)	3.22 (0.78–13.25)	0.106
	High	Positive	12 (38.7)	41 (25.5)	4.04 (1.03–15.85)	0.045
Non‐cardia	Low	Negative	7 (12.7)	47 (29.2)	1.00	
	Low	Positive	16 (29.1)	34 (21.1)	3.52 (1.28–9.65)	0.015
	High	Negative	14 (25.5)	39 (24.2)	2.63 (0.95–7.26)	0.062
	High	Positive	18 (32.7)	41 (25.5)	3.33 (1.24–8.99)	0.018

^a^ Unconditional logistic regression adjusted for age, sex, smoking, and drinking.

Besides *H. pylori* infection status, we also analyzed the joint effects between *RP11‐244 K5.6* expression and other possible influence factors on GC risk, such as smoking and drinking habits. Although the subjects with only high expression status of *RP11‐244 K5.6* or with only drinking habit showed no significant differences (both *p* > 0.05), a higher risk of GC was found in subjects with both high *RP11‐244 K5.6* expression and drinking habit (OR, 2.47, 95%CI: 1.07–5.72) compared to the never drinking subjects with low lncRNA expression (Table S2). No significant associations were found when we considered smoking habit in the combination of *RP11‐244 K5.6* expression on GC risk (all *p* > 0.05) (Table S3).

## 4 DISCUSSION

GC is one of the most common cancers worldwide. Although more comprehensive treatments for GC were developed due to the advances in technology, the prognosis of GC remains poor accounting for one of the most common causes for cancer death.^1^ Early diagnosis with effective and noninvasive biomarkers provides a major solution to improve the GC prognosis. Circulating lncRNAs in plasma were reported to be associated with various human cancers and considered as potential markers.^21^ However, systematic studies in GC on comprehensive lncRNA profiles are still needed. The present study evaluated the comprehensive GC‐associated lncRNA profiles and validated potential noninvasive lncRNA biomarkers for GC screening in a high‐risk population.

Accumulative studies have suggested that lncRNAs may serve as promising noninvasive biomarkers for cancer diagnosis with highly stable forms in plasma. A previous hospital‐based study by our collaborative team screened and validated three lncRNA biomarkers for GC diagnosis using the Arraystar Human lncRNA Microarray v3.0.^20^ In this study, we used an updated Arraystar Human lncRNA Microarray v4.0 with 40,916 lncRNAs in genome‐wide to compare the comprehensive lncRNA expression profiles in GC and control plasma samples from a high‐risk cohort. A total of 1206 differential lncRNAs were identified in GC cases with 470 lncRNAs upregulated and 736 downregulated. Additionally, the differential lncRNAs screened by the present microarray detection were further confirmed with the differential candidates found by our collaborative team.^20^ The eight consistent upregulated lncRNAs in the two studies were validated in two stages and identified *RP11‐244 K5.6* as a potential noninvasive biomarker for GC detection. Although *RP11‐244 K5.6* has been found over‐expressed in GC subjects by the two Chinese studies, no clear regulation functions were reported in carcinogenesis studies so far. Further mechanism studies are needed.

*H. pylori* infection has been proven to play a key role in gastric carcinogenesis.^16^ Many lncRNA studies in GC or gastric lesion subjects have suggested possible relationships between *H. pylori* infection and lncRNA regulation. For example, our research team has previously found significant joint effects between the expressions of *LINC00152* or *H19* with *H. pylori* infection on the risk of GC.^22^ The regulatory network studies suggested that many GC‐associated lncRNAs, which can be regulated by *H. pylori* infection, may target different signaling pathways, such as the suppression of Wnt/β‐catenin pathway by downregulation of lnc‐GNAT1‐1 after *H. pylori* infection.^21^, ^23^, ^24^ Depending on a lncRNA/mRNA analysis set from the GEO database, a study described the cross‐networks among lncRNA‐mRNA‐ceRNA and identified significant immune and differentiation function regulations in the *H. pylori*‐positive GC progression process.^24^ Additionally, lncRNAs were also found to participate in *H. pylori*‐associated carcinogenesis by targeting specific oncogenes or tumor suppressors.^25^, ^26^, ^27^, ^28^ The present study evaluated possible influence factors, including *H. pylori* infection, smoking and drinking habits, in combination with *RP11‐244 K5.6* expression on GC risk. Elevated GC risk was found for subjects with the high expression of *RP11‐244 K5.6* or subjects with *H. pylori* infection and for the subjects with both high expression and infection. However, the joint effect was not statistically confirmed between *RP11‐244 K5.6* expression and *H. pylori* infection in this study, which still need further large sample size validation.

There are two types of GC in our study including 37% cardia and 63% non‐cardia cancers. About 89% non‐cardia and 20% cardia GC cases are attributable to *H. pylori* infection.^29^ In our study, *H. pylori* infection also acted as the most important risk factor for non‐cardia GC, while no significant joint effect was found between *RP11‐244 K5.6* expression and *H. pylori* infection. Many associations have been reported on lncRNAs with cardia cancer development and metastasis, such as the tumor suppressor role of lncRNA MEG3^30^ and cancer cell proliferation and metastasis‐promoting functions of lncRNA ZFAS1.^31^ Interestingly, we found a significantly higher risk of cardia GC for those with high *RP11‐244 K5.6* expression and *H. pylori* infection status (OR, 4.04, 95%CI: 1.03–15.85) rather than the subjects with only high lncRNA expression (OR, 3.22, 95%CI: 0.78–13.25) or only *H. pylori* infection (OR, 3.55, 95%CI: 0.84–14.90) compared to *H. pylori*‐negative subjects with low lncRNA expression. Our findings preliminarily suggested potential interactions between *H. pylori* infection and lncRNA expression in cardia GC, which still need larger sample size validation and mechanism confirmation.

Our study has several strengths. Firstly, the investigation of the present study provided differential lncRNAs list of GC for potential noninvasive biomarkers in plasma compared with the invasive tissue‐originating biomarkers. Secondly, an updated comprehensive lncRNA microarray was used to screen candidate differential lncRNAs in genome‐wide, which were further confirmed by the findings of our collaborative team with a similar microarray and validated in two stages. Thirdly, the subjects enrolled in the present study were selected from an upper gastrointestinal cancer screening cohort in a high‐risk area for GC rather than from hospital, which may provide a population‐based evidence for the potential GC screening biomarker identification.

Despite the strengths of our study, limitations should also be taken into consideration. The Human LncRNA Microarray screening was conducted only in 10 pairs of GC and control plasma samples. The two‐stage validation enrolled a modest sample size with only one significant lncRNA finally identified in the present study. Further validations on more candidate lncRNAs from microarray screening are needed. In addition, the differential lncRNA, such as *RP11‐244 K5.6* in the present study, still need functional studies for the possible biological mechanism in gastric carcinogenesis.

## 5 CONCLUSION

In conclusion, this study comprehensively described GC‐associated lncRNA profiles in plasma and identified *RP11‐244 K5.6* as a potential noninvasive lncRNA biomarker. The interactions of *RP11‐244 K5.6* expression and possible influence factors, such as *H. pylori* infection, were evaluated with the risk of cardia and non‐cardia GC, respectively. Our findings preliminarily suggest that the expression of *RP11‐244 K5.6* in plasma may serve as a potential biomarker for the risk of GC, which still needs further validation.

## AUTHOR CONTRIBUTIONS

**Xiaoying Guo:** Data curation (equal); formal analysis (equal); methodology (equal); validation (equal); visualization (equal); writing – original draft (equal); writing – review and editing (equal). **Yang Zhang:** Data curation (equal); formal analysis (equal); investigation (equal); methodology (equal); project administration (equal); resources (equal); supervision (equal); validation (equal); visualization (equal); writing – original draft (equal); writing – review and editing (equal). **Zhiyi Zhang:** Project administration (equal); resources (equal); supervision (equal). **Linzhi Lu:** Project administration (equal); resources (equal); supervision (equal). **Yuqin Liu:** Project administration (equal); resources (equal); supervision (equal). **Zhexuan Li:** Project administration (equal); resources (equal); supervision (equal). **Tong Zhou:** Project administration (equal); resources (equal); supervision (equal). **Jingying Zhang:** Supervision (equal); validation (equal). **Wen‐Qing Li:** Resources (equal); supervision (equal). **Wei‐Cheng You:** Conceptualization (equal); investigation (equal); project administration (equal); resources (equal); supervision (equal); writing – original draft (equal); writing – review and editing (equal). **Guoquan Tao:** Project administration (equal); resources (equal); supervision (equal). **Wanqing Chen:** Conceptualization (equal); funding acquisition (equal); project administration (equal); resources (equal); supervision (equal); writing – original draft (equal); writing – review and editing (equal). **Hongmei Zeng:** Conceptualization (lead); data curation (equal); formal analysis (equal); funding acquisition (lead); methodology (equal); project administration (equal); resources (equal); supervision (equal); validation (equal); visualization (equal); writing – original draft (equal); writing – review and editing (equal). **Kaifeng Pan:** Conceptualization (lead); data curation (equal); funding acquisition (lead); project administration (equal); resources (equal); supervision (equal); writing – original draft (equal); writing – review and editing (equal).

## FUNDING INFORMATION

This research was supported by the National Natural Science Foundation of China (81672819, 81773538), the Beijing Natural Science Foundation (7182032), and the International (Regional) Cooperation and Exchange Project (NSFC‐DFG, 81861138041).

## CONFLICT OF INTEREST STATEMENT

The authors declare that they have no conflicts of interest.

## Supporting information


Data S1.
