# Supplementary material for: Gastric cancer‐associated long non‐coding RNA profiling and noninvasive biomarker screening based on a high‐risk population cohort
Source: Cancer Med. 2023 Apr 21;12(11):12728–38. doi: 10.1002/cam4.5905 (PMC10278487; doi:10.1002/cam4.5905)
Supplement: Supplementary file 1 — Data S1. [file CAM4-12-12728-s001.docx]

Figure S1 Stability comparison of the three reference genes in the discovery set


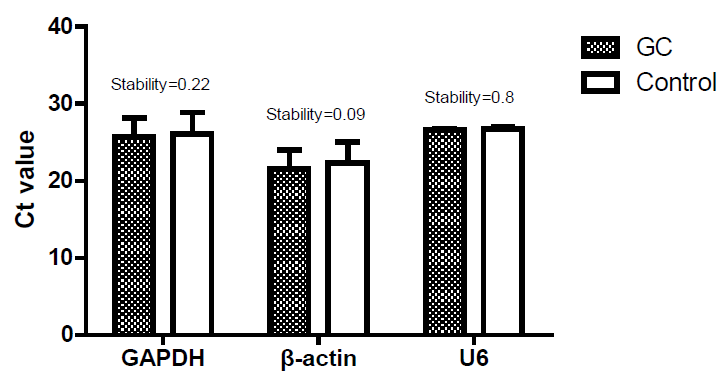


Table S1 Primer sequences for the candidate lncRNAs and reference genes

| Genesymbol | Forward (5’-3’) | Reverse (5’-3’) |
| --- | --- | --- |
| U6 | F:5’GCTTCGGCAGCACATATACTAAAAT3’ | R:5’CGCTTCACGAATTTGCGTGTCAT3’ |
| *RP11-521D12.1* | F:5' GTGGGTGCTACAGATGGTGAC 3’ | R:5’ GCAGGACCAGACATAGAAACTC 3’ |
| *AC011995.3* | F:5' GAACTCACGTCTTTGAACTTCAGTT 3’ | R:5’ CCTGTGTGACAGAAGACCATTCA 3’ |
| *RP11-5P4.3* | F:5' AGAGGTTCTGATCGCCCTTAA 3’ | R:5’ AGCTGGGAGATAGTGGTTTGC 3’ |
| *RP11-244K5.6* | F:5' CAACTTCCCAAGCAGAATGAG 3’ | R:5’ ATCCAGGTACAGAAACAAACATC 3’ |
| *RP11-422J15.1* | F:5' AGACACAAGGAGCCAAGACAG 3’ | R:5’ GGCCTTGCAGTAGAGTAGGTG 3’ |
| *CTD-2306M5.1* | F:5' GCACCATTCTCAGCAAACTAA 3’ | R:5’ GGCTTCATGTATTATTTATGAGTGA 3’ |
| *CTC-428G20.2* | F:5' TTGAAACAGAAACCCGAGAAT 3’ | R:5’ GAGTAAAGGAAAGGCAAAGTG 3’ |
| *AC009133.20* | F:5' TGAGTGCTCATGGGTTTCTACCT 3’ | R:5’ TGGGCTCCCTCTTCCTCTTCT 3’ |
| ꞵ-actin | F:5' GCCCTGAGGCACTCTTCCA 3' | R:5' CGGATGTCCACGTCACACTTC 3' |
| GAPDH | F:5' TCGACAGTCAGCCGCATCTTCTTT 3' | R:5' ACCAAATCCGTTGACTCCGACCTT 3' |

Table S2 Joint effects between *RP11-244K5.6* expression levels and drinking on risk of GC

| LncRNA  status | Drinking habit | GC Cases n(%) | Controls  n(%) | OR(95%CI) | *P*^a^ |
| --- | --- | --- | --- | --- | --- |
| Low | Never | 26(30.2) | 65(40.4) | 1.00 |  |
| Low | Ever or current | 8(9.3) | 16(9.9) | 1.30(0.48-3.48) | 0.606 |
| High | Never | 36(41.9) | 63(39.1) | 1.44(0.77-2.68) | 0.252 |
| High | Ever or current | 16(18.6) | 17(10.6) | **2.47(1.07-5.72)** | **0.035** |

^a^Unconditional logistic regression adjusted for age, sex, *H. pylori* infection and smoking.

Table S3 Joint effects between *RP11-244K5.6* expression levels and smoking on risk of GC

| LncRNA  status | Smoking habit | GC Cases n(%) | Controls  n(%) | OR(95%CI) | *P*^a^ |
| --- | --- | --- | --- | --- | --- |
| Low | Never | 17(19.8) | 36(22.3) | 1.00 |  |
| Low | Ever or current | 17(19.8) | 45(28.0) | 0.82(0.31-2.18) | 0.690 |
| High | Never | 18(20.9) | 33(20.5) | 1.15(0.50-2.64) | 0.748 |
| High | Ever or current | 34(39.5) | 47(29.2) | 1.61(0.64-4.02) | 0.310 |

^a^Unconditional logistic regression adjusted for age, sex, *H. pylori* infection and drinking.
